# Galectin-9: diverse roles in skin disease

**DOI:** 10.3389/falgy.2025.1614277

**Published:** 2025-07-16

**Authors:** Lin Wang, Chuqiao Zhang, Jiang Ji, Qingqing Jiao

**Affiliations:** ^1^ Central Research Laboratory, The First Affiliated Hospital of Soochow University, Suzhou, China; ^2^ Department of Dermatology, The Second Affiliated Hospital of Soochow University, Suzhou, China; ^3^ Department of Dermatology, The First Affiliated Hospital of Soochow University, Suzhou, China

**Keywords:** Galectin-9, Tim-3, atopic dermatitis, urticaria, systemic lupus erythematosus

## Abstract

Galectin-9 (Gal-9) is a multifunctional immunomodulatory molecule involved in cell growth, differentiation, adhesion, communication, and death. Galectin-9 mediates its physiological functions through interactions with multiple membrane receptors, including T-cell immunoglobulin mucin-domain containing-3 (Tim-3), immunoglobulin E, and the hyaluronan receptor CD44. In recent years, Gal-9 has been extensively studied in autoimmune diseases, tumor microenvironments, and viral infections. The circulating levels of this lectin demonstrate clinical correlation with disease progression in both acute and chronic infectious conditions. In addition, Gal-9 can potentially be a reliable, sensitive, and noninvasive biomarker of disease severity in many skin diseases. However, there has not been a review of Gal-9 studies in patients with dermatosis. This review summarizes recent advances in understanding Gal-9's immunomodulatory mechanisms in chronic spontaneous urticaria pathophysiology, systemic lupus erythematosus, atopic dermatitis, melanoma, systemic sclerosis, herpes simplex virus infection, bullous pemphigoid, psoriasis, vitiligo, maculopapular exanthema, and skin grafting to provide a reference for future research. Gal-9 is an important regulator of immune homeostasis whose level changes significantly in many skin diseases, and validation was performed in a mouse model using exogenous Gal-9. Ongoing studies are necessary to clarify the pathophysiology of Gal-9, identify the potential of Gal-9 as a new biomarker, and develop new therapeutic approaches for skin diseases.

## Introduction

Galectin-9 (Gal-9) is a multifunctional immunomodulatory molecule involved in cell growth, differentiation, adhesion, communication, and death (1). Different galectins with β-galactoside carbohydrate recognition domains of different structures have been identified (2). It is localized in the nucleus, cell surface, cytoplasm, and extracellular matrix (3), regulating necessary signals between growth and apoptosis (4). Mitogens, toll-like receptor agonists, and proinflammatory cytokines can increase the expression of Gal-9 (5, 6). Emerging evidence suggests that extracellular Gal-9 release may occur through exosome-mediated unconventional secretion. Its pleiotropic effects are achieved via binding to an expanding repertoire of receptors: T cell immunoglobulin and mucin domain-containing molecule 3 (Tim-3) (7), surface-expressed protein disulfide isomerase (PDI) (8), Immunoglobulin E (IgE) and Cluster of Differentiation (CD44) complexes, lysosomal-associated membrane protein-2 (LAMP2), co-stimulatory molecules (CD137/CD40), macrophage scavenger receptor CD206, immune checkpoint regulators [V-domain Ig Suppressor of T cell Activation (VISTA)/Dectin-1], and bacterial lipopolysaccharide (LPS) receptor 4-1BB—all participating in inflammatory cascades. The combination of Gal-9 and Tim-3 regulates the activation and differentiation of T cells (9). Carcinoembryonic antigen cell adhesion molecule-1 (CEACAM-1) acts as a heterotypic binding partner for TIM-3, essential for its T-cell suppressive activity (10). Through Programmed Cell Death Protein 1 (PD-1) engagement, Gal-9 counteracts Tim-3-mediated apoptotic signaling in T lymphocytes (11). In addition, by combining with Tim-3, Gal-9 promotes the maturation of dendritic cells (DCs) and mononuclear cells and the secretion of cytokines (12, 13). Gal-9 also binds to CD44, which is expressed in various cell types, thereby inhibiting type 2 cells recruitment and reducing the accumulation of activated lymphocytes and eosinophils in inflammatory lesions (14). Gal-9 binds to Death Receptor 3 (DR3) to promote the activity of regulatory T cells (Tregs) (15). By binding CD40, Gal-9 inhibits the proliferation and induces the apoptosis of T cells (16). Gal-9 binds to 4-1BB to transform T-cell-related signals and regulates the functional activities of T cells (17). By binding to VISTA, Gal-9 promotes T-cell apoptosis (18). Gal-9 binds Dectin-1, acts on macrophages, and participates in tolerogenic macrophage programming and adaptive immune suppression (19). By binding to CD206, it drives tumor tissue angiogenesis and the production of chemokines (20).

**Figure 1:**
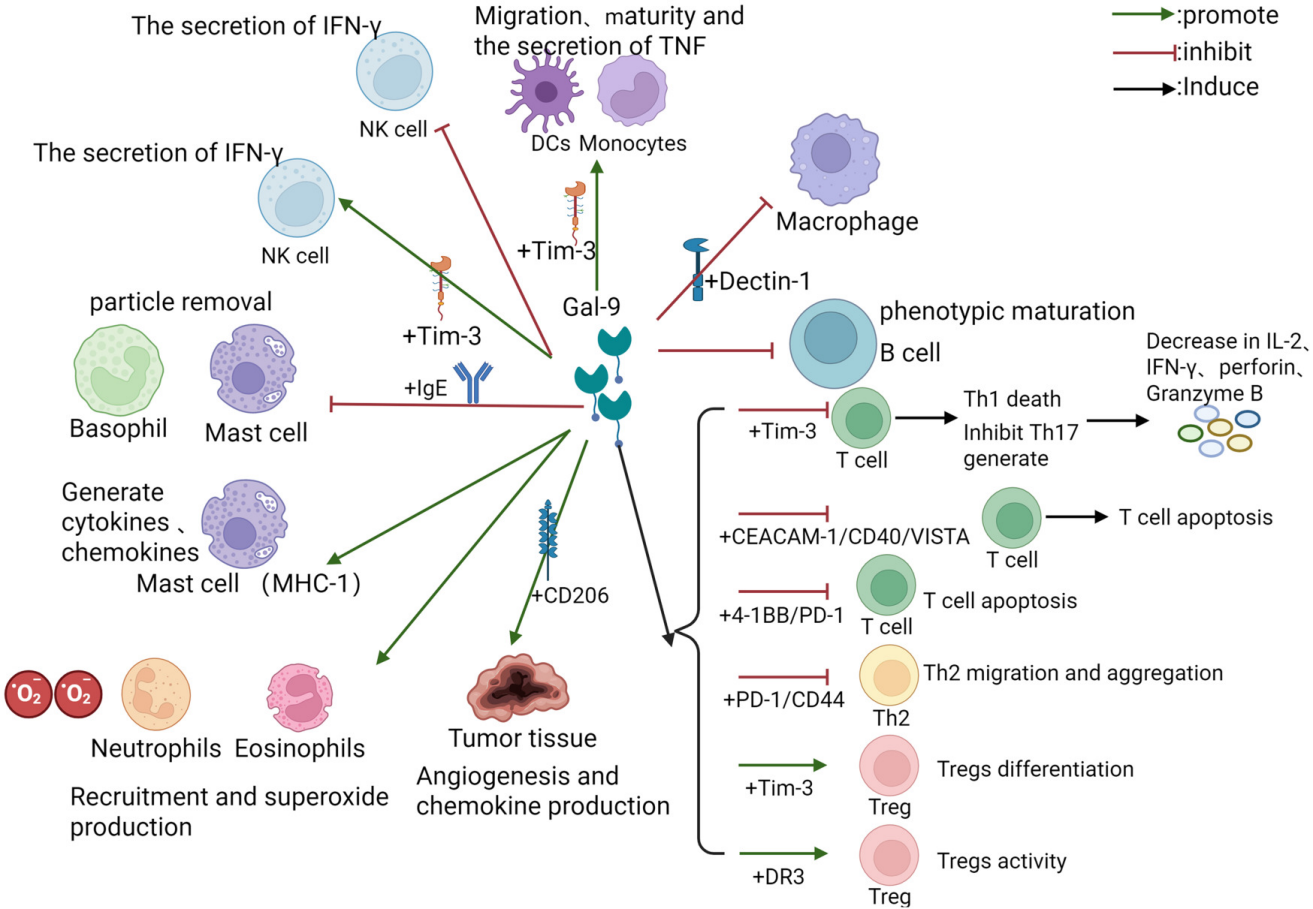
The regulatory pattern of Gal-9 on diverse immune cells. The figure depicts the regulation of diverse immune cells by Gal-9, including the involved receptor pathways, related cytokines and inflammatory processes. The green arrow represents facilitation, the red arrow represents inhibition, and some downstream effects are marked behind the black arrow. Abbreviations: Gal-9, Galectin-9; Tim-3, T cell immunoglobulin and mucin domain-containing molecule 3; Th, T helper; IL, Interleukin; TNF, Tumor necrosis factor; IFN, Interferon; Treg, Regulatory T cell; DC, Dendritic cell; CEACAM, carcinoembryonic antigen cell adhesion molecule; NK, Natural Killer; IgE, Immunoglobulin E; CD, cluster of differentiation; VISTA, V-domain Ig Suppressor of T cell Activation; CD137, 4-1BB; PD-1, Programmed Cell Death Protein 1; DR, death receptor.

Gal-9 is involved in many processes of the immune cell-mediated immune response (Figure 1). By encoding the LGALS9 gene (3), Gal-9 is widely expressed on the surface of thymocytes and controls T-cell fate (21). In T cells, Gal-9 induces the death of type 1 cells by binding to Tim-3 (22), inhibits the generation of T helper 17 (Th17) cells (23), and promotes the induction of Tregs (24), contributing to the maintenance of the Th17/Treg balance (25). When Gal-9 is knocked out in experimental mice, Tregs decrease (26). Gal-9 promotes the Transforming growth factor-β (TGF-β) signaling pathway, promotes the expression of FOXP3, and strengthens the differentiation of Tregs (27). Gal-9 inhibits the cytotoxic activity of type 1 cells and CD8^+^ T cells by promoting Treg cells, and the binding of CD8^+^ T cells to Gal-9 leads to T-cell exhaustion and reduced secretion of Interferon-γ (IFN-γ) and Tumor necrosis factor-α (TNF-α) (28, 29). Pharmacological inhibition of the Gal-9/Tim-3 axis enhances T-cell proliferative capacity and restores effector molecule production [IFN-γ, Interleukin-2 (IL-2), perforin, granzyme B] (18). While sparing Tim-3-negative type 2 cells from apoptosis, Gal-9 facilitates type 2 cells' migration via PDI interaction (8). Concentration-dependent effects are observed: high-dose Gal-9 triggers caspase activation in type 1/type 2 cells independent of Tim-3, whereas low doses stimulate Tim-3-independent cytokine secretion (IFN-γ/TNF-α) (30). However, there have been no definitive threshold doses, and the dose fluctuates according to the different microenvironments of different diseases. Gal-9 reverses Human Immunodeficiency Virus latency by promoting CD4^+^ T cells, inhibits the memory of CD8^+^ T cells, and weakens the antiviral response (31). Gal-9 functions by terminating the immune response to alleviate inflammation, as mentioned above.

However, a dual effect was observed in the mast cell/basophilic cell line. On the one hand, Gal-9 binding to IgE can block the formation of IgE/Ag complexes, thereby inhibiting mast cell/basophil degranulation (24, 32). In addition, Gal-9 inhibits excessive mast cell degranulation and prevents passive cutaneous anaphylaxis in mice (33). Mast cell studies reveal Gal-9's IgE-independent activation of Human Mast Cell-1 (HMC-1) cells, inducing proinflammatory mediator release (32). It demonstrates broad immunoregulatory capacity through B-cell signaling suppression (34), promotion of B-lymphocyte/macrophage apoptosis, and Tim-3-independent inhibition of Natural Killer (NK) cells IFN-γ production.

Gal-9 is also an eosinophil chemoattractant that triggers the signaling cascade required for innate immune activation (4), recruits eosinophils and neutrophils to the site of infection, contributes to superoxide production (35), and assists in dendritic cell (DC) maturation (13, 36). The maturation of DCs is a critical step in initiating the immune response. Gal-9 promotes DC maturation by upregulating the expression of costimulatory molecules such as CD40, CD54, and HLA-DR (13). Gal-9 also promotes the cell migration process of DCs. Gal-9 promotes the secretion of TNF-α by monocytes via Tim-3 receptors and enhances innate immunity (12), which is involved in the killing of gram-negative bacteria (37).

Gal-9 protects against autoimmune diseases by promoting the timely shutdown of adaptive immunity (38, 39). The anti-metastatic potential of Gal-9 involves dual mechanisms: impairment of circulating tumor cell extravasation and inhibition of extracellular matrix adhesion processes (40, 41). High expression of Gal-9 is associated with tumor colony formation (42, 43). Increased levels of Gal-9 have been found in many diseases and are associated with disease severity (44, 45). Gal-9 can potentially serve as a reliable, sensitive, and non-invasive biomarker of disease severity (3). Additionally, Gal-9 can mediate various immune responses in individuals with dermatosis and has a corresponding channel or evidence related to the onset of skin disease. Therefore, in this review, we review the role of Gal-9 in skin disease.

## Role of Gal-9 in skin diseases

### Chronic spontaneous urticaria

Chronic spontaneous urticaria (CSU) is defined as the appearance of itchy wheals and/or angioedema for longer than 6 weeks. The cell‒cell interactions among mast cells, basophils, and eosinophils/T cells regulate their function and may involve the CSU pathomechanism. In addition to the second generation of antihistamines (46), the latest therapies based on pathological mechanisms include omalizumab, dupilumab, and Bruton's tyrosine kinase inhibitors (47). According to the pathological mechanism, it is necessary to explore the development of new drugs to relieve patients' pain since there is no way to achieve a radical cure for CSU. Gal-9 plays a role in type 2 cells mediated eosinophilic allergic diseases, mainly by interacting with eosinophils (35). The polarization toward type 2 immunity of Gal-9 is usually accompanied by increased production of IgG1 and IgE and the activation of eosinophils and mast cells to release cytokines and chemokines, which further exacerbates disease activity in patients with refractory CSU (48). Recently, we reported that the number of circulating Gal-9^+^ eosinophils and basophils is significantly increased in CSU patients and that the number of Gal-9^+^ cells in skin lesions is increased. Upregulation of Gal-9 is associated with disease activity, IgE levels, and negative basophil activation test (BAT) results in CSU patients. TNF-α upregulated the level of Gal-9 in eosinophils via PI38K in the CSU. Gal-9 levels are increased in patients who respond to omalizumab treatment, and omalizumab reduces Gal-9 levels in CSU patients (49). The Gal-9^+^ eosinophil/basophil ratio can be used to predict individual reactivity to omalizumab in CSU patients. Omalizumab inhibits mast-cell degranulation by reducing Fc*ε*RI expression by binding free IgE, but Gal-9 can also bind IgE directly. Whether there is dual regulation here requires further study. Patients who responded to omalizumab treatment instead had higher Gal-9 before treatment, suggesting the potential presence of a feedback loop—a high IgE environment induces upregulation of Gal-9 as a compensatory mechanism. It is necessary to distinguish between membrane-bound Gal-9 (which directly inhibits effector cells) and soluble Gal-9 (biomarker).

The negative correlation between Gal-9^+^ eosinophils/basophils and Tim-3^+^ Th17 cells in the CSU supports the above Gal-9/Tim-3 inhibition of Th17 cell generation. In addition, the serum levels of soluble Gal-9 were similar in CSU patients and HCs in our study (49). In another study, although the serum Gal-9 level was greater than that in HCs, it was not associated with sex, age, disease duration, Chronic Urticaria Quality of Life Questionnaire score, CSU activity (Urticaria Activity Score Over 7 Days, UAS7 and Sabroe's grade), or autologous serum skin test (ASST) activity (50). These findings suggest that soluble Gal-9 may be a bystander molecule. Notably, this study did not stratify patients by ASST status or autoantibody profiles, potentially diluting subtype-specific associations. This heterogeneity underscores the need for endotype-driven biomarker analysis in future studies. Thus, different forms of Gal-9 are involved in the disease process of CSU in diverse manners: positive and bystander. However, the role of Gal-9 in CSU development requires further research stratified by autoimmune status, IgE autoantibody levels, and response to omalizumab.

### Systemic lupus erythematosus

Systemic lupus erythematosus (SLE) is a multisystem chronic autoimmune disease that is more common in women of childbearing age. T cells secrete proinflammatory cytokines, induce autoantibody production by B cells, and maintain disease via autoreactive memory T cells, leading to abnormal proportions and functions of some T-cell subsets in SLE patients (51). For example, decreased IL-2 in SLE patients leads to impaired Treg development and function and reduces the restriction of the proinflammatory factor IL-17. The pathological activation pathways linked to B-lymphocyte dysregulation encompass Toll-like receptor (TLR) signaling, β-cell activating factor (BAFF) stimulation, and B-cell receptor (BCR)-dependent activation. This multifaceted signaling dysregulation culminates in systemic loss of immune tolerance in SLE pathogenesis (52). Mehta et al. measured serum and urine Gal-9 and C-X-C motif chemokine ligand 10 (CXCL-10) levels in 97 SLE patients via ELISA. Serum Gal-9 can be used to determine the activity of SLE but has little significance in distinguishing active renal disease from active nonrenal disease. Gal-9 performed slightly better than the validated marker CXCL-10 (53, 54). The expression of Tim-3 and the level of Gal-9 in the CD4^+^ and CD8^+^ T cells of SLE patients were also greater than those in healthy controls (54–56). Gal-9 suppresses functional maturation of plasmacytoid dendritic cells (pDCs) and B lymphocytes, impairing their TLR7/TLR9 ligand-induced cytokine responses. Mechanistically, it inhibits both immune complex-triggered and neutrophil extracellular trap-mediated pDC activation (57). Furthermore, Gal-9 demonstrates pathway inhibition through mTOR/p70S6K signaling- a critical regulator of TLR-dependent IFN secretion in pDCs and autoantibody generation in B cells- via CD44 interaction-mediated suppression of these cellular populations. Gal-9 is a good indicator of disease activity in children with SLE, and larger samples are needed for further experimental confirmation (57). Enhancing coinhibitory signaling to block immune responses may be helpful in the treatment of autoimmune diseases (58). Enhancing co-inhibitory signaling to block immune responses may be helpful in the treatment of autoimmune diseases (59). Further studies revealed that monocyte immune responses in the peripheral blood of SLE patients were inhibited by a Gal-9-blocking antigen. Tim-3 may be a new target for the treatment of SLE (60). In the future, different biomarkers will be combined to evaluate SLE more effectively (61).

It is important to note that the proposed therapeutic blockade of the Gal-9/Tim-3 axis in SLE appears to contradict its anti-inflammatory roles observed in conditions like psoriasis or HSV infection (sections below). This discrepancy highlights the context-dependent functionality of Gal-9. In SLE, Gal-9's predominant interaction within the dysregulated immune milieu, particularly its potent suppression of pDC-derived IFN-α via mTOR/p70S6K signaling and potential engagement with other receptors like VISTA (Table 1), contributes to the breakdown of tolerance and perpetuates autoimmunity. Therefore, in this specific pathological context characterized by aberrant innate immune activation and interferon signatures, inhibiting Gal-9 signaling is proposed as a strategy to restore immune balance.

**Table 1:** Galectin-9 receptors, downstream effects, and roles in skin diseases.

Receptor	Downstream effects	Molecular mechanisms	Associated skin diseases	Role in disease context
Tim-3	T-cell apoptosis/exhaustion; Th17 suppression	Caspase activation; FOXP3 upregulation; mTOR/p70S6K-mediated pDC IFN-α suppression	Psoriasis, HSV, SLE, CSU	Anti-inflammatory: Psoriasis/HSV (limits pathogenic T cells)
CD44	Blocks hyaluronan adhesion; Enhances NK activity	Suppresses chemokine release; Activates NK clonal expansion	Melanoma, Allergic asthma	Pro-tumor: Melanoma (NK-mediated antitumor) Anti-inflammatory: Asthma (reduces eosinophilia)
IgE	Inhibits mast cell degranulation	Blocks IgE-antigen complex formation	CSU	Anti-inflammatory: Prevents histamine release
CD206	Chemokine secretion; Angiogenesis	Activates tumor-associated macrophages	Melanoma, SSc	Pro-tumor: Supports tumor microenvironment
VISTA	T-cell exhaustion; pDC IFN-α suppression	Inhibition of TLR7/9 via mTOR/p70S6K; Caspase activation	SLE, Autoimmune disorders	Pro-inflammatory: Drives loss of tolerance (SLE)
4-1BB	Modulates T-cell signaling	Stabilizes costimulatory signals to control viral latency	HSV infection	Anti-inflammatory: Limits CD8+ T-cell hyperresponse
Dectin-1	Tolerogenic macrophage programming	Suppresses innate immune activation	Tumor microenvironment (Melanoma)	Pro-tumor: Immune evasion
PD-1	Counteracts Tim-3-induced apoptosis	Synergistic checkpoint inhibition	Melanoma, Psoriasis	Anti-tumor: Restores T-cell function
DR3	Treg activation	Enhances TGF-β signaling	Skin graft rejection	Anti-inflammatory: Prolongs graft survival
PDI	Promotes T-cell migration	Regulates redox environment	CSU, Viral infections	Bidirectional: Immune activation vs. pathogen entry
CD40	Inhibits T-cell proliferation; Induces apoptosis	Caspase-dependent apoptosis	Autoimmune diseases	Anti-inflammatory: Suppresses aberrant T-cell responses

Abbreviations: AD, atopic dermatitis; CSU, chronic spontaneous urticaria; SLE, systemic lupus erythematosus; SSc, Systemic sclerosis; HSV, herpes simplex virus; Treg, regulatory T cell; TLR, Toll-like receptor; CCL11, C-C motif chemokine ligand 11.

### Atopic dermatitis

Atopic dermatitis (AD), is the most common form of skin inflammation and is characterized by itching, peeling, and sometimes damp red skin. Owing to damage to cutin formation from the terminal differentiation of cells, AD barrier dysfunction in skin lesions leads to a high incidence of bacterial, fungal, or viral infection (62). AD, a type 2-skewed inflammatory dermatosis, manifests peripheral eosinophilia and mast cell hyperactivation (63). Elevated Gal-9 levels in AD microenvironments regulate inflammatory cascades and keratinocyte hyperproliferation (28). Serological profiling revealed Gal-9/Tim-3 axis-mediated suppression of type 1/Th17 responses with concomitant type 2/Th22 polarization (64). Notably, Gal-9 modulates keratinocyte immunoreactivity by attenuating IL-6/IL-17 release from IL-4-activated cells and IL-6 production in TNF-α/IFN-γ-stimulated models (65). Clinical investigations demonstrate Gal-9 serum elevation positively correlating with SCORAD indices and lesional surface area in AD (9). The serum level of Gal-9 is higher in patients with AD and positively correlated with the severity index and eczema area (63, 66). Gal-9 expression was observed in mast cells and epidermal keratinocytes in skin lesions (65). Immunohistochemical analyses detect Gal-9 expression in cutaneous mast cells and activated keratinocytes, supporting its biomarker potential (3, 66). Post-therapeutic reduction of epidermal and circulating Gal-9 levels implies pro-inflammatory activity (63). Kim et al. reported that probiotic-induced Gal-9 could alleviate dinitrochlorobenzene-induced AD in mice, demonstrating the great potential of Gal-9 in the treatment of AD (67). Murine studies indicate exogenous Gal-9 exerts anti-inflammatory properties through selective suppression of IFN-γ/IL-17 production without altering type 2 cytokine profiles (68). This evidence suggests that Gal-9 is a relevant therapeutic target.

### Melanoma

Melanoma, also known as skin cancer, is a malignant tumor derived from melanin cells (69). Early diagnosis can improve the survival rate of melanoma patients, so more sensitive biomarkers and targeted combined immunotherapy targets need to be developed. Studies have shown that in patients with stage IV melanoma, Gal-9 expression is associated with a relatively high survival rate (70). Although the assessment of Gal-9 expression is not currently part of routine clinical practice for melanoma staging or prognostication, this association highlights its potential as a novel prognostic biomarker. High expression of Gal-9 promotes the growth of tumors, including promoting tumor angiogenesis (20). Gal-9 may promote NK cell-mediated antitumor effects via amplification of macrophages (71). Tumor immunology studies reveal CD44-associated clonal expansion of Gal-9^+^ NK cells in melanoma-bearing mice (72). Mechanistically, Gal-9 impedes melanoma-endothelial adhesion and extracellular matrix remodeling (75). Gal-9 expression inversely correlates with metastatic potential: high levels in primary lesions/nevi vs. low expression in metastases (42), demonstrating direct pro-apoptotic effects on melanoma cells. It has been reported that Gal-9 can directly promote the apoptosis of melanoma cells (73). The expression of Gal-9 is high in primary melanoma lesions and nevi, and low in metastatic melanoma lesions (42). This suggests that the Gal-9 overall may be a tumor suppressor. The Gal-9/Tim-3 axis critically mediates cytotoxic T lymphocyte (CTL) exhaustion in melanoma immunity. Experimental Gal-9 knockdown combined with Tim-3 blockade enhances antitumor responses by preventing CTL depletion (74). Complementary to molecular inhibition, Ren et al. recently demonstrated that a tumor-targeted nanodrug (FSGG/siGal-9) combined with photothermal therapy enhances Gal-9 blockade efficacy, promoting cytotoxic T-cell recruitment and melanoma regression *in vivo*. The dual role of Gal-9 (pro-tumorigenic in the microenvironment vs. tumor-suppressive via direct apoptosis and immune modulation) and its association with survival underscore its complexity and the need for further investigation to determine its precise clinical utility. Notably, solid tumors exhibit predominant Gal-9 expression among immune checkpoints. Preferentially expressed antigen in melanoma (PRAME)—a poor-prognosis tumor antigen—co-regulates multiple checkpoint molecules (PD-1/PD-L1/Gal-9) through epigenetic silencing effects (76). Further validation in larger cohorts and the development of standardized assays for Gal-9 detection in tumor tissue or liquid biopsies are necessary steps towards evaluating its potential integration into clinical decision-making algorithms or as a target for novel immunotherapies.

### Systemic sclerosis

Systemic sclerosis (SSc) represents a multi-organ autoimmune disorder marked by progressive cutaneous/organ fibrosis and microvascular pathology (77). Current therapeutic strategies involving cyclophosphamide, mycophenolate mofetil, and hematopoietic stem cell transplantation show efficacy in dermal sclerosis improvement. Nintedanib and tocilizumab were temporarily not found to effectively relieve skin hardening effects (78). As a biomarker, Gal-9 is inferior in sensitivity and specificity to chemokine cytokine ligand 18 (CCL18) (79). The combination of multiple biomarkers can improve the efficiency of disease detection. The cytotoxic T lymphocyte-associated antigen 4 fusion protein demonstrates antifibrotic activity in SSc models (80). Pathological Gal-9 overproduction in SSc patients suppresses type 1 cytokine generation through CD4^+^ T cell modulation (81). Dermal fibroblasts facilitate Treg-to-type 2 cells conversion while secreting Gal-9 to inhibit IFN-γ expression in infiltrating lymphocytes, thereby promoting fibrotic progression (81). Elevated serum Gal-9 levels in diffuse/limited SSc subtypes correlate with erythrocyte sedimentation rate, mortality risk, and visceral involvement (82). Both Tim-3 overexpression and serum elevation exhibit positive associations with cutaneous induration severity, while PD-1/Tim-3 co-expression participates in SSc pathogenesis (83, 84).

### Herpes simplex virus infection

Herpes simplex virus (HSV) infections predominantly manifest as mucocutaneous lesions, with atypical presentations including eczema herpeticum in atopic dermatitis, herpes gladiatorum in athletes, and disseminated eruptions in Darier/Sézary syndromes (85). Erythema multiforme is common in HSV infection. Currently, the main treatment for herpes simplex virus is acyclovir, and drug resistance is relatively low (86). However, HSV vaccine development, gene therapy, and various pathological mechanisms are still under further study. Some studies have shown that Gal-9 promotes recovery from HSV infection by reducing type 1 and CD4^+^ T cells, increasing the expression of anti-inflammatory factors such as TGF-β and IL-10, and downregulating the expression of proinflammatory factors such as IFN-γ and IL-6 (87). Gal-9 can stabilize 4-1BB and control innate immunity to the virus (17). In an animal model of HSV infection, Gal-9-deficient mice presented a stronger CD8^+^ cell response. Moreover, blockade of Gal-9 also reduced the response of Tregs. Intraperitoneal injection of Gal-9 reduces CD8^+^ cell responses (88, 89). Gal-9 participates in blocking CD8^+^ T cells in HSV infection after incubation (89). These findings suggest that blocking Gal-9 may facilitate better and faster clearance of HSV.

### Bullous pemphigoid

Bullous pemphigoid (BP) is an autoimmune disease with antibodies against the dermal‒epidermal adhesion complex, and type 2 cytokines and IL-17 are highly expressed in the peripheral blood and skin (90–92). In autoimmune bullous dermatoses (AIBDs), Immunophenotypic analysis reveals upregulated PD-1/Tim-3 expression in lesional skin, particularly within CD8^+^ T lymphocytes and macrophages (93). Beyond eosinophil cationic protein (ECP) (94), Gal-9 emerges as a prognostic biomarker for BP severity, potentially mediating eosinophil chemotaxis critical to blister formation (95). Monocyte-derived Gal-9 facilitates granulocyte recruitment in BP pathogenesis, with elevated expression observed in epidermal keratinocytes of BP lesions and intestinal epithelia in type 2 immunity-associated food allergies (96). Gal-9 expression was significantly observed in epidermal keratinocytes of BP-affected skin and intestinal epithelial cells of patients with type 2 immunity-associated food allergy (96). The main goal of BP treatment is to control the development of new skin lesions, and a treatment plan needs to be formulated according to different patients (97). Gal-9 and its interaction with mononuclear cells remain to be elucidated.

### Psoriasis

Psoriasis vulgaris, a chronic immune-mediated dermatosis, features hyperproliferative keratinocytes and psoriasiform plaques (98). Dysregulated Gal-9/Tim-3 signaling contributes to type 1 cells/Th17 imbalance, with experimental Gal-9 administration reducing pathogenic T cell populations (IFN-γ^+^/IL-17^+^) and attenuating epidermal hyperplasia (68, 99). Although lesional Tim-3^+^ CD8^+^ T cells/macrophages are prevalent, the Gal-9/PD-L1 dissociation suggests predominant type 2 immunity pathway involvement (93). While some studies propose Gal-9 as a therapeutic target, clinical data from Nofal et al. show no significant correlation between serum Gal-9 levels and Psoriasis Area Severity Index, though associations with leukocyte/eosinophil counts and hepatic enzymes persist (66, 100). Gal-9 is found to be elevated in patients with psoriasis and may become a new therapeutic target (68, 101). Plasma Gal-9 level was not correlated with psoriasis severity score but was correlated with white blood cell, eosinophil, and ALT levels, indicating that its Gal-9 can reflect the severity of the disease (101). At present, there are still some patients who do not respond to biological agents or have secondary treatment failure; that is, the response to the original effective biological agents has decreased. Moreover, the current price of biological agents is high, and the development of new therapeutic targets is still very promising (102).

### Vitiligo

The loss of functional melanocytes in the skin or hair and the appearance of white macules. Vitiligo is a common acquired pigmentary disease (103). Vitiligo is fully reversible by suppressing autoimmunity and by promoting the regeneration of a stem-cell niche for melanocytes in hair follicles. Considering the high recurrence rate, more effective targeted therapies are needed (104). Vitiligo pathogenesis involves CD8^+^ T cell-mediated melanocyte destruction through IFN-γ-chemokine axis activation (105). Elevated Tim-3/Gal-9 expression in peripheral blood and peri-lesional skin correlates with depigmentation extent, suggesting their involvement in melanocyte targeting. Co-upregulation of Tim-3/PD-1 on cytotoxic T lymphocytes indicates checkpoint-mediated immune dysregulation, positioning these molecules as potential biomarkers and immunotherapeutic targets (106). CXCL9, a T cell chemoattractant, reliably reflects vitiligo disease activity (105).

### Maculopapular exanthema

Maculopapular exanthema (MPE) is involved in immediate allergic drug reactions, and type 1 cytokines and CD4^+^ T cells play important roles (107). Immunophenotypic analysis revealed type 1 lymphocyte Tim-3 downregulation in malignant pleural effusion (MPE) patients. Exogenous recombinant Gal-9 administration induced Treg expansion concomitant with type 1 population contraction. Type 1 cell levels were restored after Tim-3 blockade (26).

### Skin graft status

Acute allograft rejection pathophysiology involves proinflammatory type 1/Th17 cell axis activation. The expression of endogenous Gal-9 is associated with the severity of rejection, but exogenous Gal-9 has the opposite effect on graft rejection (108, 109). Gal-9/Tim-3 pathway engagement exerts dual immunoregulatory effects: suppressing CD4^+^/CD8^+^ alloreactive T cell responses through IFN-γ suppression, thereby enhancing allogeneic skin graft survival (110). Importantly, Tim-3 antibody blockade neutralizes donor-derived Treg-mediated graft tolerance in preclinical models (111).

## Conclusion and future perspectives

Gal-9 exhibits paradoxical pro- and anti-inflammatory effects across skin diseases, which may be determined by three interconnected contextual factors: receptor expression profiles, microenvironmental cues, and concentration gradients.

Gal-9's function depends on its binding partners in specific microenvironments. *Anti-inflammatory role*: Binding to Tim-3 on Th1/Th17 cells induces apoptosis or exhaustion (e.g., in psoriasis and HSV infection), while interaction with IgE inhibits mast cell degranulation in urticaria. *Pro-inflammatory role*: Engagement with CD44 or CD206 promotes chemokine secretion and angiogenesis in melanoma and SSc, whereas ligation to VISTA exacerbates T-cell apoptosis in autoimmunity. Cytokine milieus influence Gal-9 activity. In type 2-dominant diseases (e.g., AD, BP), Gal-9 amplifies eosinophil recruitment and keratinocyte hyperproliferation via IL-4/IL-13-driven pathways. Conversely, in type 1-skewed conditions (e.g., SLE), it suppresses IFN-α production by pDCs through mTOR inhibition (57). Oxidative stress and post-translational modifications further modulate its functionality. Low-dose Gal-9 enhances T-cell migration and cytokine secretion, while high concentrations induce caspase-dependent apoptosis of activated T cells (112). This dose-dependent effect explains its divergent roles in localized or systemic inflammation.

The seemingly paradoxical recommendation to block Gal-9/Tim-3 in SLE while exploring its therapeutic potential in AD or its anti-inflammatory effects in psoriasis/HSV exemplifies this contextual complexity. In SLE, the dominant pathological consequence of Gal-9 signaling (e.g., suppression of protective IFN-α by pDCs, promotion of T cell exhaustion/dysfunction via VISTA or other partners) outweighs any potential regulatory benefits, warranting inhibition. Conversely, in Th1/Th17-driven conditions like psoriasis or certain viral settings like HSV, Gal-9's ability to induce apoptosis in pathogenic T cells or promote regulatory pathways via Tim-3 or other receptors becomes therapeutically desirable. This underscores that the net immunomodulatory effect of Gal-9 is not intrinsically pro- or anti-inflammatory but is determined by the specific disease pathophysiology and the dominant signaling pathways it engages within that context.

Given the above, we propose the following model. Gal-9 acts as an immune rheostat that amplifies or suppresses inflammation based on disease-specific receptor landscapes, cytokine environments, and spatiotemporal concentration gradients. Therapeutic strategies should thus target *contextual partners* (e.g., Tim-3 blockade in SLE, Gal-9 delivery in AD) rather than Gal-9 alone.

This lectin demonstrates context-dependent immunomodulation—serving as immune checkpoint in hyperactive states while potentiating immune responses during immunosuppression (28). Its bidirectional effects manifest through cell-type specific actions and microenvironmental influences (1), with functional outcomes determined by disease pathophysiology, administration protocol, and concentration gradients (39). The pharmacokinetics of Gal-9 are clear in mice after subcutaneous or intraperitoneal injection (9). This finding is beneficial for further therapeutic studies of Gal-9. The sensitivity or specificity of Gal-9 in some of the abovementioned skin diseases has surpassed that of existing clinically recognized biomarkers, and some exogenous Gal-9 or Gal-9 blocking agents have been proven to be helpful in the treatment of *in vitro* or animal conditions. Under the current treatment framework, the response of some refractory skin disease patients to previous drugs has decreased, and related molecules, such as Gal-9, deserve further study to identify more effective and biochemically comprehensive treatments. Gal-9 emerges as a viable noninvasive biomarker for multimodal disease monitoring when integrated with clinical parameters. Therapeutic strategies combining anti-Gal-9/Tim-3 biologics with complementary checkpoint inhibitors show promise in next-generation immunotherapy development (113). However, the reasons for the low affinity and activity of Gal-9 need to be solved, such as research on targeted delivery methods (28). To overcome challenges like low tissue-specificity and rapid clearance, novel Gal-9 delivery systems are emerging. Nanoparticle-based platforms enable targeted transdermal delivery, while Gal-9-Fc fusion proteins enhance stability and receptor engagement. Exosome-mediated Gal-9 cargo and viral vector-driven gene therapy further offer precision in modulating localized immune responses. Future work should prioritize disease-contextual design—such as Tim-3-targeted delivery for Th1-driven pathologies or CD44-focused systems for atopic dermatitis—to harness Gal-9's dual roles therapeutically.

In summary, Gal-9 has dynamic expression patterns across various dermatological conditions, exhibiting both proinflammatory and immunoregulatory effects depending on the disease context. Current evidence suggests that its dual functionality manifests through T-cell modulation, cytokine network regulation, and keratinocyte interactions, although the precise mechanisms involved remain incompletely elucidated. While Table 1 highlights the consistent dysregulation of Gal-9 in psoriasis, atopic dermatitis, and cutaneous malignancies, its biomarker potential requires careful disease-specific evaluation because of its paradoxical roles in different pathologies. Particularly compelling is its prognostic correlation with disease severity in autoimmune dermatoses, supporting further investigation into therapeutic targeting. However, technical challenges in detection standardization and tissue-specific isoform analysis currently limit its clinical translation as a standalone biomarker. Future studies should prioritize longitudinal monitoring and mechanistic dissection to establish context-dependent clinical applications for Gal-9 in dermatological practice.
